# Evaluation of a prospective interdisciplinary assessment of return to play in male professional rugby union following lower-limb injury: A pilot study

**DOI:** 10.1016/j.jsampl.2025.100115

**Published:** 2025-08-11

**Authors:** Molly F. McCarthy-Ryan, Stephen D. Mellalieu, Holly S.R. Jones, Adam Bruton, Isabel S. Moore

**Affiliations:** a Cardiff School of Sport and Health Sciences, Cardiff Metropolitan University, Cardiff, UK; b College of Health, Medicine and Life Sciences, Brunel University London, London, UK

**Keywords:** Lower-limb functional rehabilitation pathway, Team sport, Injury and prevention, Biomechanics, Psychology

## Abstract

The return-to-play process is multifactorial, requiring input from multiple disciplines for rehabilitation. This pilot study used a prospective interdisciplinary approach to assess male professional Rugby Union players' (n ​= ​7) rehabilitation following a non-contact lower-limb injury. Kinetic and self-efficacy assessments were conducted across three rehabilitation phases (acute, middle, late). Biomechanical changes (p ​< ​0.05) were observed across all phases; alongside self-efficacy increases. Moderate-to-strong positive relationships (r ​= ​0.77–0.80) were found between kinetic and self-efficacy changes. Practitioners should incorporate both measures throughout rehabilitation, as each offers distinct insights into recovery despite their high correlation. An interdisciplinary approach ensures a comprehensive assessment, enhancing players’ rehabilitation outcomes.

## 1 Introduction

Rugby Union is associated with a high incidence of contact and non-contact injuries, particularly lower-limb injuries, which make up the majority of match and training injuries [1]. Following lower-limb injury, athletes often adjust their movement strategy by producing different forces to maintain performance or to compensate for the injured limb, potentially increasing the risk of subsequent and or recurrent injury [2,3]. Although kinetic assessments have traditionally been concentrated at the point of return-to-play (RTP), there is increasing recognition of their value throughout rehabilitation. Limiting assessments to the point of RTP constrains the prospective monitoring of movement strategies [4]. Commonly employed kinetic assessments at the point of RTP include the countermovement jump [2], drop jump [[5], [6], [7]], and the lateral hurdle hop as a surrogate to assess change of direction [8,9]. These assessments are selected for their capacity to evaluate discrete aspects of lower limb biomechanics, such as stretch-shortening cycle efficiency, landing mechanics, and movement proficiency [[10], [11], [12]]. While observational methods such as video analysis are often used in practice, kinetic assessments offer objective and reliable measures of movement quality, though the research in their clinical application remains limited.

Self-efficacy, a key psychological predictor of behaviour change, plays a central role in an athlete's recovery from injury and is positively associated with rehabilitation adherence and limb function [13,14]. Although self-efficacy is typically assessed at the point of RTP [13,15] it represents an internal cognitive appraisal of one's ability to execute behaviours necessary to achieve specific outcomes [16], and thus reflects an ongoing psychological response to the rehabilitation process. Measuring self-efficacy throughout the rehabilitation process, rather than solely at RTP, allows for a comprehensive understanding of the athlete's psychological readiness and behavioural engagement during recovery. An interdisciplinary approach, integrating biomechanical and psychological factors, is therefore essential in rehabilitation, as it is fundamental to optimising RTP outcomes [[17], [18], [19]]. This reflects the principles of the biopsychosocial model, which recognises the dynamic interaction between physical, psychological, and social domains throughout the recovery process [17]. An interdisciplinary approach, integrating biomechanical and psychological factors, is therefore essential in rehabilitation. Fundamental to optimising RTP outcomes, as it reflects the principles of the biopsychosocial model, which recognises the dynamic interaction between physical, psychological, and social domains in recovery. Despite consensus that RTP is a shared decision [19], there is limited research on monitoring these factors throughout the rehabilitation process. This study aimed to pilot an interdisciplinary approach to characterise Rugby Union players' rehabilitation response following a non-contact lower-limb injury.

## 2 Methods

### 2.1 Participants

Seven male professional Rugby Union players (height 1.80 ​± ​0.02 ​m; mass 100.3 ​± ​11.4 ​kg; age 24 ​± ​4 years) from a professional team in Wales participated over one full season. All participants had sustained a non-contact lower-limb injury (≥14 days-lost). To overcome the fundamental issues of the small sample size due to resource constraints [20], statistical power was met at 0.8, with the effect size statistics used to assess the relevance of differences between testing sessions. All players had sustained a minimum of 14-days’ time loss non-contact lower-limb injury [21]. Injury details were collected and reported by the medical performance manager as part of a wider injury surveillance project. Each of the seven participants received a clear injury diagnosis, confirmed through clinical assessment and, where appropriate, supported by diagnostic imaging (e.g., x-ray), surgical intervention, or specialist consultation. All diagnoses were verified by qualified medical professionals to ensure accuracy. The injured body areas were knee: 57 ​% (n ​= ​4), hip: 29 ​% (n ​= ​2), and ankle: 14 ​% (n ​= ​1), with 57 ​% (n ​= ​4) being ligament injuries and 43 ​% (n ​= ​3) muscle injuries. Ethical approval was obtained from the University Institutional Review Board. Pre-injury baseline data were collected during pre-season on both limbs, but only injured limbs were measured following an injury.

### 2.2 Phases of RTP

Following injury, players entered a three-phase rehabilitation pathway (acute, middle, late). A lower-limb functional rehabilitation pathway (Supplementary Fig. 1) was developed using the biopsychosocial model with input from the Rugby team's medical staff. The mean ​± ​SD duration spent in each rehabilitation phase was 7 ​± ​4 weeks for the acute phase, 10 ​± ​5 weeks for the middle phase, and 6 ​± ​2 weeks for the late phase. Median durations (95 ​% CI) were 4 weeks (3–11) for the acute phase, 10 weeks (1–19) for the middle phase, and 6 weeks (2–10) for the late phase. All players in this study received individualised rehabilitation programmes, designed by the medical performance manager, each being progressive and considerate of their specific rehabilitation needs. Progression through the rehabilitation phases were not uniform across players but were determined functionally, based on individual readiness and clinical presentation. A shared decision-making approach was adopted, involving input from a multidisciplinary team including medical, strength and conditioning, and coaching staff [19]. This collaborative approach ensured that progression through the RTP process considered a range of biopsychosocial risk factors, as well as the athlete's ability to tolerate increased load and complexity of activity [18].

### 2.3 Data collection

Pre-injury baseline data were collected from all team players during the pre-season. Following an injury, players entered a structured rehabilitation pathway consisting of three phases, acute, middle, and late each comprising progressively functional movements (Supplementary Fig. 1). Objective measures of unilateral static postural control were measured using PASCO dual axis force plates (PS-2142; 1000 ​Hz) in the acute phase. PASCO single axis force plates (PS-2141; 1000 ​Hz) were used for the dynamic objective measurements performed in the middle and late phases (unilateral drop jump and lateral hurdle hop, respectively). Full methodological details, including measurement protocols and setup, are provided in the Supplementary File 1.

Reliability and validity of the static unilateral postural control, unilateral drop jump and lateral hurdle hop on the PASCO forces plates were examined. Detailed methods and results can be found in Supplementary File 1.

### 2.4 Self-efficacy assessment

A bespoke 17-item self-efficacy questionnaire assessed rehabilitation perceptions across task, injury, and social dimensions following Bandura's [22] guidelines using an 11-point Likert scale (0–100; Supplementary Table 1). The questions were worded for all sub-dimensions as ‘On a scale of 0 (totally disagree) to 100 (totally agree), please rate your level of agreement with the following statements about your current levels of confidence’. Face validation (using the teams' physiotherapists and players) of the questionnaire was found to be 100 ​% suitable for assessing all aspects of RTP. The questionnaire evidenced high internal consistency, with an excellent Cronbach's alpha score of >0.96 for each item recorded for the data collected from all participants across data collection points. The questionnaire was completed immediately following biomechanical assessment in all three RTP phases. Players were tested weekly to determine their readiness to progress to the next stage of the rehabilitation programme.

### 2.5 Data analysis

A 4th order, recursive low-pass Butterworth filter (cut-off frequencies determined by residual analyses: 35 ​Hz for postural control, 25 ​Hz for dynamic movements) was applied and kinetic variables calculated (Matlab R2019b). Sway path was calculated for the acute phase as the total distance of the CoP trajectory [23]. Each 20 ​s trial was broken into 4 ​× ​5 ​s intervals [24]. For the dynamic the variables of interest were ground contact time (s), net impulse (BW·s), take-off velocity (m·s^−1^), initial peak landing force (BW), initial instantaneous loading rate landing (BW·s^−1^), flight time (s) and jump height (m). For self-efficacy, mean item scores were computed for each of the three sub-dimensions and the full questionnaire.

### 2.6 Statistical analysis

Kinetic and self-efficacy variables were calculated as mean ​± ​SD, with normality assessed using the Shapiro–Wilk test. Following confirmation of measurement reliability (see details in Supplementary File 1 and Supplementary Table 2), contrast analyses were conducted to compare responses between the objective pre-injury baseline and the final session of each rehabilitation phase. Paired t-tests or Wilcoxon signed-rank tests were used to compare data within phases, depending on normality. Given the small sample size, Hedges' g was used to determine the magnitude of change, and relative change (RC) was calculated between the first and last session of each phase. Effect sizes were interpreted as small (g ​= ​0.2–0.5), medium (g ​= ​0.51–0.8), and large (g ​> ​0.8) [25]. To explore associations between biomechanical and psychological responses, correlations were calculated using the relative change from the first to last session for each phase. Pearson's r or Spearman's rho (r_s_) were used as appropriate, with correlation strength interpreted as weak (r ​< ​0.3), moderate (r ​= ​0.4–0.6), or strong (r ​> ​0.6) [26]. Only variables that showed statistically significant changes across each RTP phase and demonstrated at least a moderate correlation were reported. All analyses were conducted using SPSS (v27.0), with significance set at p ​< ​0.05.

## 3 Results

### 3.1 Kinetics

During the acute phase of postural control assessment, a significant reduction in eyes-open sway path was observed from the first to the final session (p ​= ​0.02, g ​= ​0.44; Table 1). However, this change did not exceed the minimal detectable difference (MDD), as detailed in Supplementary File 1 and Supplementary Table 2. In contrast, the eyes-closed condition showed a significantly larger sway path when comparing the pre-injury baseline to the final session (p ​= ​0.004, g ​= ​0.75), with the increase surpassing the MDD threshold.

**Table 1:** Mean ​± ​SD of pre-injury baseline, first and last testing session of the acute middle and late rehabilitation phase and relative change (RC) between testing session comparison.

			Postural Control Eyes Open				Postural Control Eyes Closed		
				Baseline – Last	First – Last			Baseline – Last	First – Last
				RC%	RC%			RC%	RC%
Sway path (m)	Pre-injury baseline		0.17 ​± ​0.04	21 ​%			**0.41 ​± ​0.10**	20 ​%**^+^**	
	Rehabilitation	First session	**0.22** ​± ​**0.07**		17 ​%		0.52 ​± ​0.10		13 ​%
		Last session	0.19 ​± ​0.06				0.52 ​± ​0.13		
			Drop jump				Lateral hurdle hop		

Ground contact time (s)	Pre-injury baseline		0.36 ​± ​0.09	18 ​%**^+^**			**0.35** ​± ​**0.10∗∗**	20 ​%**^+^**	
	Rehabilitation	First session	**0.43** ​± ​**0.06**∗		35 ​%**^+^**		0.27 ​± ​0.04		5 ​%
		Last session	0.32 ​± ​0.02				0.28 ​± ​0.04		
Net impulse (BW·s)	Pre - injury baseline		0.59 ​± ​0.10	47 ​%			**0.49** ​± ​**0.13∗∗**	33 ​%	
	Rehabilitation	First session	0.44 ​± ​0.18		17 ​%**^+^**		**0.31** ​± ​**0.07∗**		29 ​%**^+^**
		Last session	0.50 ​± ​0.16				0.43 ​± ​0.06		
Take-off velocity (m·s^−1^)	Pre-injury baseline		1.91 ​± ​0.78	27 ​%**^+^**			1.76 ​± ​0.41	23 ​%	
	Rehabilitation	First session	**1.28** ​± ​**0.23**∗		41 ​%**^+^**		1.33 ​± ​0.71		13 ​%
		Last session	2.27 ​± ​0.78				1.53 ​± ​0.47		
Initial peak landing force (BW)	Pre-injury baseline		2.50 ​± ​0.25	13 ​%			2.33 ​± ​0.44	83 ​%	
	Rehabilitation	First session	**1.66** ​± ​**0.41**∗		32 ​%**^+^**		**3.10** ​± ​**0.61∗**		69 ​%**^+^**
		Last session	2.45 ​± ​0.84				2.52 ​± ​0.38		
Initial instantaneous loading rate landing (BW·s^−1^)	Pre-injury baseline		51.98 ​± ​7.50	50 ​%			**405.35** ​± ​**88.83∗∗**	40 ​%**^+^**	
	Rehabilitation	First session	50.39 ​± ​14.89		71 ​%		460.34 ​± ​200.48		56 ​%
		Last session	46.33 ​± ​19.89				579.29 ​± ​143.03		
Flight time (s)	Pre-injury baseline		0.32 ​± ​0.04	7 ​%			0.27 ​± ​0.03	11 ​%	
	Rehabilitation	First session	**0.23** ​± ​**0.06**∗		28 ​%**^+^**		0.25 ​± ​0.04		4 ​%
		Last session	0.32 ​± ​0.05				0.24 ​± ​0.04		
Jump height (m)	Pre-injury baseline		0.13 ​± ​0.03	14 ​%			–		
	Rehabilitation	First session	**0.07** ​± ​**0.03**∗		48 ​%**^+^**				
		Last session	0.12 ​± ​0.04						

*Note*: Bold indicates *p* ​≤ ​0.05, or hedges g ​≥ ​0.80 ∗ and dashed underlined indicates significant difference between the first and last session. ∗∗ and underlined indicates significant difference between pre-injury baseline and the last session. ​+ ​Indicates changes were greater than the minimal detectable change, Supplementary Table 2 contains full detail.

During unilateral drop jumps in the middle phase, ground contact time significantly decreased (p ​< ​0.001, g ​= ​2.28), while take-off velocity (p ​= ​0.03, g ​= ​1.55), peak landing force (p ​< ​0.001, g ​= ​0.39), flight time (p ​< ​0.001, g ​= ​1.40), and jump height (p ​< ​0.001, g ​= ​1.38) all showed significant increases. Each of these changes exceeded the MDD, with full details provided in Supplementary File 1 and Supplementary Table 2.

In the late phase, during lateral hurdle hops, net impulse increased (p ​= ​0.04, g ​= ​1.58), though this change did not exceed the MDD (Supplementary Table 2). Conversely, peak landing force significantly decreased (p ​= ​0.02, g ​= ​1.00), surpassing the MDD threshold. When compared to the pre-injury baseline, ground contact time (p ​= ​0.03, g ​= ​0.96) and net impulse (p ​= ​0.02, g ​= ​1.49) both decreased, while instantaneous loading rate increased (p ​= ​0.04, g ​= ​1.09); all of these changes were greater than the MDD, full details are available in Supplementary File 1 and Supplementary Table 2.

### 3.2 Self-efficacy

Self-, task- and injury-efficacy showed improvement across all phases (acute, middle, late; Table 2). These improvements were significant for overall self-efficacy (p ​= ​0.04, g ​= ​1.94; p ​< ​0.001, g ​= ​2.63; p ​= ​0.008, g ​= ​1.27), task-efficacy (p ​= ​0.03, g ​= ​2.12; p ​< ​0.001, g ​= ​2.63; p ​= ​0.008, g ​= ​1.31), and injury-efficacy (p ​= ​0.02, g ​= ​1.46; p ​< ​0.001, g ​= ​2.20). No changes were found in social efficacy.

**Table 2:** Mean ​± ​SD of first and last testing session of the acute, middle and late rehabilitation phase and relative change (RC) between testing session comparison.

			Postural control		Drop jump		Lateral hurdle hop	
			First – Last		First – Last		First – Last	
				RC%		RC%		RC%
Overall self-efficacy	Rehabilitation	First session	**70** ​± ​**10∗**	7 ​%	**70** ​± ​**6∗**	18 ​%	**78** ​± ​**6∗**	11 ​%
		Last session	78 ​± ​9		90 ​± ​5		88 ​± ​8	
Task-efficacy	Rehabilitation	First session	**71** ​± ​**13∗**	9 ​%	**68** ​± ​**9∗**	21 ​%	**75** ​± ​**3∗**	11 ​%
		Last session	80 ​± ​9		86 ​± ​8		85 ​± ​9	
Injury-efficacy	Rehabilitation	First session	**69** ​± ​**6∗**	10 ​%	**70** ​± ​**11∗**	23 ​%	**76** ​± ​**9∗**	12 ​%
		Last session	80 ​± ​11		91 ​± ​4		88 ​± ​10	
Social-efficacy	Rehabilitation	First session	87 ​± ​12	4 ​%	90 ​± ​14	8 ​%	86 ​± ​11	8 ​%
		Last session	90 ​± ​10		98 ​± ​6		94 ​± ​9	

*Note*: Bold indicates *p* ​≤ ​0.05, or hedges g ​≥ ​0.80 ∗ and dashed underlined indicates significant difference between the first and last session.

### 3.3 Interdisciplinary assessment

Moderate-to-strong relationships were found between the RC of eyes-open sway path and overall self-efficacy (r ​= ​0.45, p ​= ​0.35) and task-efficacy (r ​= ​−0.61, p ​= ​0.15). In the middle phase, strong relationships were observed between overall self-efficacy and ground contact time and flight time (r ​= ​−0.70, p ​= ​0.11; r ​= ​0.80, p ​= ​0.03 respectively), task-efficacy (r ​= ​−0.66, p ​= ​0.11; r ​= ​0.87, p ​= ​0.01 respectively), and injury-efficacy (r ​= ​−0.60, p ​= ​0.18; r ​= ​0.77, p ​= ​0.04 respectively). Take-off velocity and initial peak landing force had a strong correlation between their RC and injury-efficacy (r ​= ​0.60, p ​= ​0.17; r_s_ ​= ​0.79, p ​= ​0.03 respectively). In the late phase, net impulse was associated with overall self-efficacy (r ​= ​0.56, p ​= ​0.25), and peak landing force with task-efficacy (r ​= ​−0.60, p ​= ​0.20).

## 4 Discussion

This study aimed to pilot an interdisciplinary approach to characterise Rugby Union players’ rehabilitation response after a non-contact lower-limb injury.

In the acute phase, postural control improved between the first and final sessions, likely due to rehabilitation interventions targeting somatosensory deficits and neural plasticity [27]. However, deficits persisted during eyes-closed assessments, a more complex task than eyes-open. In the middle phase, jumping performance improved, with increased jump heights. Shorter ground contact times and improved performance suggest more efficient stretch-shortening cycle use or alternative movement strategies [28]. Larger landing forces may reflect greater jump heights or prioritisation of performance over safe landing mechanics. In the late phase, reduced peak force and vertical impulse were observed. Larger forces in the initial session may reflect unpreparedness for multiplanar movements like lateral hurdle hops [2]. Despite late-phase improvements, altered movement strategies persisted, indicated by greater instantaneous loading rates. These findings suggest rehabilitation must address biomechanical and sensory adaptations throughout phases to reduce injury risk, prioritising safe landing.

Overall, self-, task-, and injury-efficacy improved during all three phases. High self-efficacy has been shown to enhance athletes’ adherence to rehabilitation, and is associated with improvements in pain perception, athletic identity, and limb function [29]. As players achieve greater biomechanical improvements they simultaneously use their gained mastery experience to internalise these improvements, concomitant in higher self-efficacy [13,16]. Social-efficacy has been shown to mitigate identity disruption and dissatisfaction during rehabilitation [30]. In our study, it remained consistently high across all phases, potentially due to athletes maintaining a strong sense of team affiliation. Practitioners should consider including each efficacy dimension to profile players and intervene as appropriate during rehabilitation.

Guided by the lower-limb functional rehabilitation pathway, associations between kinetic performance and self-efficacy emerged across all rehabilitation phases. A strong positive relationship was identified between the RC in peak landing force and injury-efficacy, as well as flight time and overall self-, task-, and injury-efficacy. These associations were most evident for variables such as flight time where performance feedback can be obtained [29], rather than mechanical variables (e.g., instantaneous loading rate). A recursive relationship likely exists, whereby biomechanical improvements enhance self-efficacy, which in turn bolsters performance [16], fostering a positive perception of athletes' capabilities [31].

This study highlights the critical interplay between biomechanical and psychological factors in the lower limb injury rehabilitation. Significant improvements in self-, task-, and injury-efficacy emphasise integrating physical and psychological assessments emphasising the necessity of integrating both physical and psychological assessments into rehabilitation protocols. These enhancements were particularly pronounced when performance feedback was provided, underscoring its role in fostering efficacy and optimising recovery outcomes. The findings advocate for a holistic, interdisciplinary approach to rehabilitation, ensuring that athletes recover physical function and develop confidence in their abilities to successfully return to sport.

## Funding

Funding from the Knowledge Economy Skills Scholarship (KESS) via the European Social Fund (Welsh Government).

## Declaration of competing interest

The authors declare the following financial interests/personal relationships which may be considered as potential competing interests: Dr Molly Frances McCarthy-Ryan reports financial support was provided by Knowledge Economy Skills Scholarship (KESS) via the European Social Fund (Welsh Government). If there are other authors, they declare that they have no known competing financial interests or personal relationships that could have appeared to influence the work reported in this paper.
