# Supplementary material for: Evaluation of a prospective interdisciplinary assessment of return to play in male professional rugby union following lower-limb injury: A pilot study
Source: JSAMS Plus. 2025 Aug 11;6:100115. doi: 10.1016/j.jsampl.2025.100115 (PMC13008437; doi:10.1016/j.jsampl.2025.100115)
Supplement: Multimedia component 1 [file mmc1.docx]

Supplementary file 1

**METHODS**

Biomechanical assessments

In the acute phase, static unilateral postural control was assessed under two conditions: eyes-open and eyes-closed. Each trial was 20 seconds in duration, interspersed with 30 second rest between trials. Players were instructed to stand on their injured limb, place their hands on their hips, flex their contralateral non weight bearing limb at the knee and to look straight ahead for both conditions. Players were informed that should they come out of this starting position; they should regain it as soon as possible as the trial would not be stopped.

During the middle phase players performed a unilateral drop jump from 20 cm. Players were instructed to stand upright with their hands on their hips, step off the block from their injured limb, and once they hit the floor to jump as high as they can and spend as little time as possible on the force plate. Players had 30 seconds rest between trials.

A lateral hurdle hop was used to assess the late phase. Players were required to hop unilaterally over a 15 cm hurdle and immediately hop back to their initial starting position. If testing their right leg, players were instructed to stand on their right foot to the left of the hurdle (on the left force plate), with the first hop being in a rightwards direction over the hurdle, and the second hop back in a leftwards direction to the original starting position. Each trial was interspersed with 1-minute rest periods.

Statistical analysis

Prior to analysing the main study outcomes, the reliability of the PASCO force plates was evaluated. Relative test-retest reliability was quantified using intraclass correlation coefficients (ICC_(2,k)_) with 95% confidence intervals [1], and interpreted as poor (0–0.49), moderate (0.50–0.74), good (0.75–0.89), or excellent (0.90–1.00) [2]. Absolute reliability was assessed using the Standard Error of Measurement (SEM) and the Minimal Detectable Difference (MDD) with 95% confidence intervals [3,4], to quantify measurement precision.

**RESULTS**

Test-retest reliability

Test-retest reliability analyses indicated moderate reliability for postural control during the acute phase, with ICC_(2,k)_ values of 0.81 for the eyes-open condition and 0.95 for the eyes-closed condition (Supplementary Table 2). These results were further supported by SEM values ranging from 0.02 to 0.04 for the eyes-open and eyes-closed conditions, respectively. The MDD ranged from 0.07 to 0.11 for the eyes-open and eyes-closed conditions, respectively, indicating acceptable measurement precision and the ability to detect true changes beyond measurement error.

For the drop jump during the middle phase, excellent reliability was observed, with ICC_(2,k)_ values ranging from 0.94 to 0.96. SEM values were low, ranging from 0.01 to 23.31, while MDD values ranged from 0.04 to 40.10 (Supplementary Table 2).

In contrast, the lateral hurdle hop during the late phase demonstrated moderate reliability, with ICC_(2,k)_ values ranging from 0.50 to 0.97. SEM values varied between 0.01 and 27.31, while MDD values ranged from 0.02 to 75.71. These findings support the consistency and interpretability of the kinetic measures employed in this study.
