# Supplementary figures and images for: Evaluation of a prospective interdisciplinary assessment of return to play in male professional rugby union following lower-limb injury: A pilot study
Source: JSAMS Plus. 2025 Aug 11;6:100115. doi: 10.1016/j.jsampl.2025.100115 (PMC13008437; doi:10.1016/j.jsampl.2025.100115)

Supplementary Figure 1:Lower-limb functional rehabilitation framework.


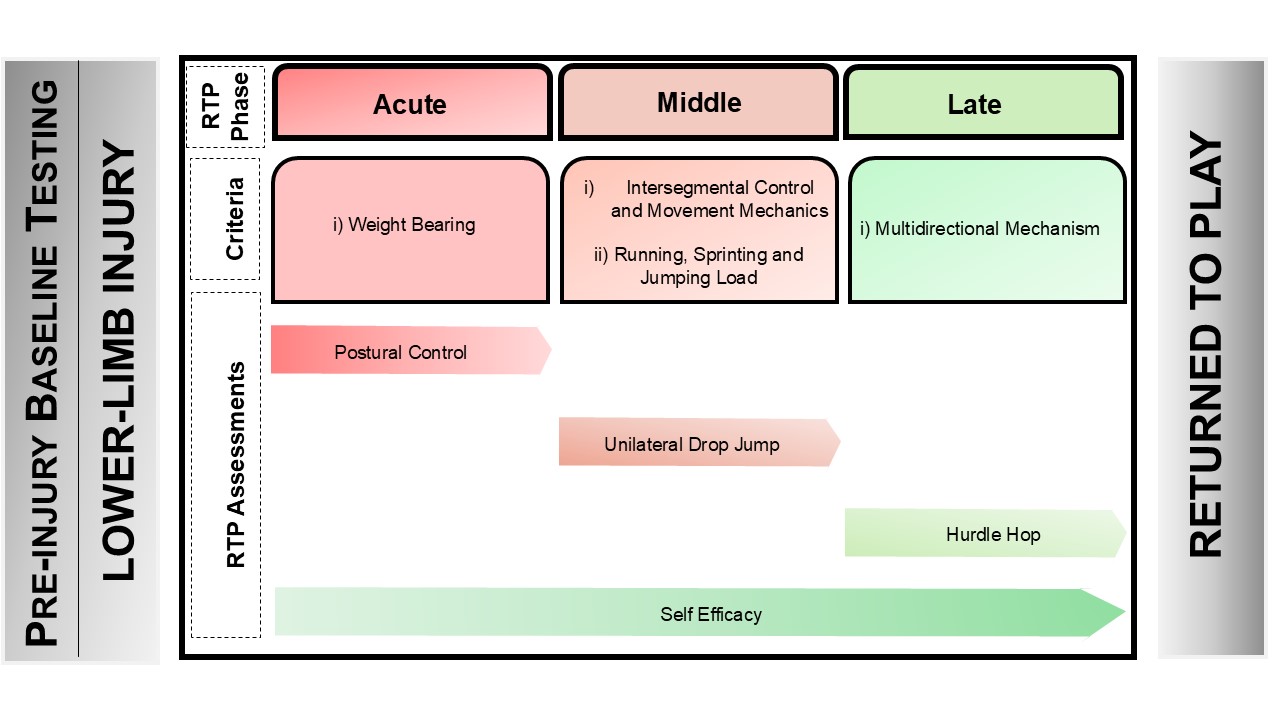

Supplement: Multimedia component 2 [file mmc2.docx]
