# Supplementary material for: Evaluation of a prospective interdisciplinary assessment of return to play in male professional rugby union following lower-limb injury: A pilot study
Source: JSAMS Plus. 2025 Aug 11;6:100115. doi: 10.1016/j.jsampl.2025.100115 (PMC13008437; doi:10.1016/j.jsampl.2025.100115)
Supplement: Multimedia component 3 [file mmc3.docx]

| Table 1 comprise of the self-efficacy questions which were devised form the self-efficacy questionnaire. With questions being divided into efficacy dimensions. | |
| --- | --- |
| All questions started with ‘On a scale of 0 (totally disagree) to 100 (totally agree), please rate your level of agreement with the following statements about your current levels of confidence | |
| Task-efficacy |  |
| I am confident I will be able to physically perform to the best of my ability when returning to sport following my injury | |
| I am confident I will be able to technically perform to the best of my ability following my injury | |
| I am confident I will be able to tactically perform to the best of my ability following my injury | |
| I am confident I will be able to make decisions to the best of my ability during training following my injury | |
| I am confident I will be able to make decisions to the best of my ability during matches following my injury | |
| I am confident I will be able to perform to the best of my ability when playing for the Ospreys following my injury | |
| I am confident I will be motivated to attend the all-testing sessions | |
| I am confident I will be able to perform the testing movements to the best of my ability | |
| Injury-efficacy |  |
| I am confident in the ability of my injured limb to perform the test movement(s) | |
| I am confident I will be ready to perform in training following my injury | |
| I am confident I will be ready to perform in matches following my injury | |
| I am confident I will be able to manage any pain caused by my injured limb | |
| I am confident I will be able to maintain performance levels by managing any pain caused by my injured limb | |
| Social-efficacy |  |
| I am confident in my ability to successfully interact with others to perform my role within the team | |
| I am confident in my ability to maintain effective on-field relationships with my teammates | |
| I am confident in my ability to maintain effective off-field relationships with my teammates | |
| I am confident in my ability to maintain effective relationships with the support staff | |
