# Supplementary material for: Evaluation of a prospective interdisciplinary assessment of return to play in male professional rugby union following lower-limb injury: A pilot study
Source: JSAMS Plus. 2025 Aug 11;6:100115. doi: 10.1016/j.jsampl.2025.100115 (PMC13008437; doi:10.1016/j.jsampl.2025.100115)
Supplement: Multimedia component 4 [file mmc4.docx]

| Supplementary Table 1 Test–retest reliability of postural control, unilateral vertical jumps, and lateral jumps measured using the PASCO force plate | | | | | | |
| --- | --- | --- | --- | --- | --- | --- |
|  | **Variables** | **Session 1** | **Session 2** | **ICC (95% CI)** | **SEM** | **MDD** |
| **Sway path (m)** | | | | | | |
|  | Eyes-open sway path (m) | 0.23±0.05 | 0.22±0.06 | 0.81 (0.56-0.92)** | 0.02 | 0.07 |
|  | Eyes-closed sway path (m) | 0.64±0.18 | 0.67±0.18 | 0.95 (0.87-0.98)*** | 0.04 | 0.11 |
|  | **Drop Jump** | | | | | |
|  | Ground contact time (s) | 0.31±0.06 | 0.31±0.05 | 0.93 (0.84-0.97)*** | 0.01 | 0.04 |
|  | Net impulse (BW·s) | 0.57±0.08 | 0.58±0.09 | 0.96 (0.91-0.98)*** | 0.02 | 0.06 |
|  | Take-off velocity (m∙s-1) | 1.28±0.27 | 1.31±0.22 | 0.86 (0.68-0.94)** | 0.09 | 0.25 |
|  | Peak landing force (BW) | 2.31±0.56 | 2.38±0.56 | 0.93 (0.85-0.97)*** | 0.15 | 0.41 |
|  | Instantaneous loading rate (BW·s-1) | 99.66±34.62 | 91.37±25.70 | 0.77 (0.48-0.90)** | 14.47 | 40.10 |
|  | Flight time (s) | 0.30±0.05 | 0.31±0.04 | 0.74 (0.39-0.89)** | 0.02 | 0.06 |
|  | Jump height (m) | 0.12±0.03 | 0.12±0.03 | 0.76 (0.44-0.90)** | 0.02 | 0.05 |
|  | **Lateral Hurdle Hop** | | | | | |
|  | Ground contact time (s) | 0.28±0.04 | 0.28±0.04 | 0.97 (0.92-0.99)*** | 0.01 | 0.02 |
|  | Net impulse (BW·s) | 0.29±0.08 | 0.29±0.08 | 0.87 (0.70-0.95)** | 0.03 | 0.08 |
|  | Take-off velocity (m∙s-1) | 1.31±0.13 | 1.29±0.14 | 0.50 (-0.19-0.79)* | 0.10 | 0.26 |
|  | Peak landing force (BW) | 2.29±0.43 | 2.25±0.40 | 0.74 (0.39-0.90)* | 0.21 | 0.59 |
|  | Instantons loading rate landing (BW·s^-1^) | 398.80±140.54 | 462.14±132.59 | 0.96 (0.91-0.98)*** | 27.31 | 75.71 |
|  | Flight time (s) | 0.26±0.03 | 0.26±0.03 | 0.80 (0.51-0.91)** | 0.01 | 0.04 |
|  | ICC: intraclass correlation_(2,k)_, SEM: standard error measurement, MDD: minimal detectable difference. * moderate ICC, ** good ICC and *** excellent ICC. | | | | | |
